# Effect of nitrogen reduction combined with biochar application on soda saline soil and soybean growth in black soil areas

**DOI:** 10.3389/fpls.2024.1441649

**Published:** 2024-09-20

**Authors:** Bo Xu, Hongyu Li, Qiuju Wang, Quanfeng Li, Yan Sha, Chen Ma, Aizheng Yang, Mo Li

**Affiliations:** ^1^ Key Laboratory of Efficient Use of Agricultural Water Resources of Ministry of Agriculture and Rural Affairs of the People′s Republic of China, Northeast Agricultural University, Harbin, China; ^2^ School of Water Conservancy and Civil Engineering, Northeast Agricultural University, Harbin, China; ^3^ National Key Laboratory of Smart Farm Technology and System, Northeast Agricultural University, Harbin, Heilongjiang, China; ^4^ International Cooperation Joint Laboratory of Health in Cold Region Black Soil Habitat of the Ministry of Education, Northeast Agricultural University, Harbin, Heilongjiang, China; ^5^ Research Center for Smart Water Network, Northeast Agricultural University, Harbin, Heilongjiang, China; ^6^ State Key Laboratory of Black Soils Conservation and Utilization, Northeast Institute of Geography and Agroecology, Chinese Academy of Sciences, Changchun, Jilin, China; ^7^ Heilongjiang Province Black Soil Protection and Utilization Research Institute, Heilongjiang Academy of Agricultural Sciences, Harbin, Heilongjiang, China; ^8^ School of Public Administration and Law, Northeast Agricultural University, Harbin, Heilongjiang, China

**Keywords:** biochar, nitrogen use efficiency, soda saline soils, soybeans, yield, water use efficiency, photosynthesis, water relations

## Abstract

The combination of biochar and nitrogen (N) fertilization in agricultural salt-affected soils is an effective strategy for amending the soil and promoting production. To investigate the effect of nitrogen reduction combined with biochar application on a soda saline soil and soybean growth in black soil areas, a pot experiment was set up with two biochar application levels, 0 (B0) and 4.5 t/hm2 (B1); two biochar application depths, 0-20 cm (H1) and 0-40 cm (H2); and two nitrogen application levels, conventional nitrogen application (N0) and nitrogen reduction of 15% (N1). The results showed that the application of biochar improved the saline soil status and significantly increased soybean yield under lower nitrogen application. Moreover, increasing the depth of biochar application enhanced the effectiveness of biochar in reducing saline soil barriers to crop growth, which promoted soybean growth. Increasing the depth of biochar application increased the K+ and Ca2+ contents, soil nitrogen content, N fertilizer agronomic efficiency, leaf total nitrogen, N use efficiency, AN, Tr, gs, SPAD, leaf water potential, water content and soybean yield and its components. However, the Na+ content, SAR, ESP, Na+/K+, Ci and water use efficiency decreased with increasing biochar depth. Among the treatments with low nitrogen input and biochar, B1H1N1 resulted in the greatest soil improvement in the 0-20 cm soil layer compared with B0N0; for example, K+ content increased by 61.87%, Na+ content decreased by 44.80%, SAR decreased by 46.68%, and nitrate nitrogen increased by 26.61%. However, in the 20-40 cm soil layer, B1H2N1 had the greatest effect on improving the soil physicochemical properties, K+ content increased by 62.54%, Na+ content decreased by 29.76%, SAR decreased by 32.85%, and nitrate nitrogen content increased by 30.77%. In addition, compared with B0N0, total leaf nitrogen increased in B1H2N1 by 25.07%, N use efficiency increased by 6.7%, N fertilizer agronomic efficiency increased by 32.79%, partial factor productivity of nitrogen increased by 28.37%, gs increased by 22.10%, leaf water potential increased by 27.33% and water content increased by 6.44%. In conclusion, B1H2N1 had the greatest effect on improving the condition of saline soil; it not only effectively regulated the distribution of salt in soda saline soil and provided a low-salt environment for crop growth but also activated deep soil resources. Therefore, among all treatments investigated in this study, B1H2N1 was considered most suitable for improving the condition of soda saline soil in black soil areas and enhancing the growth of soybean plants.

## 1 Introduction

Currently, the global population is close to 7.5 billion, and it is expected to reach 9.5 billion by 2050 (McGuire, 2015). As the population continues to grow, the demand for food continues to increase, putting continued pressure on global food supply (Heydari, 2024). The impact of climate change on soil salinization cannot be ignored. As the global temperature rises, evaporation from the soil increases, especially in arid and semi-arid regions, and water evaporation is accelerated, leading to higher soil salt concentrations, thus exacerbating soil salinization (Hassani et al., 2021). In addition, irregular or low rainfall can prevent the soil from receiving enough fresh water to wash it away, thus allowing salts to accumulate in the top layer of the soil (Slama et al., 2020). Also, the occurrence of extreme climatic events, such as droughts and floods, can further exacerbate salinization (Kumar et al., 2022).

Currently, global soil salinization is still showing an upward trend, the total area of saline-alkaline land in the world reached 954 million ha, while the total area of saline-alkaline land in China has also reached 110 million ha, which accounts for about 1/10 of the world’s saline-alkaline land (Negacz et al., 2022), and in the northeast black soil zone, there are nearly 3.7 million ha of low and medium yielding fields affected by salinization and saline wastelands only in the central and western songnen plains (Yang et al., 2022). Soil salinization in the black soil area not only causes waste of black soil resources, expansion of agricultural surface pollution, and imbalance of soil nutrient ratios (Zhang et al., 2022c), but also is prone to secondary salinization phenomenon, which leads to increased soil salinization and low nitrogen utilization, and seriously restricts the sustainable development of agriculture in the black soil area (Li et al., 2022). As an important land resource, saline soils are a “potential breadbasket” for crop production (Stille et al., 2011). Presently, increasing soil salinization and low N use efficiency are major constraints to the sustainable development of agriculture in soda saline soils (Mukhopadhyay et al., 2021). Fertilization is an effective measure for improving the fertility and crop yield in saline soils (Liu et al., 2023). However, irrational fertilization not only results in the wastage of resources, causes agricultural surface pollution, and leads to an imbalance in soil nutrient ratios but also leads to the accumulation of soil salt and secondary salinization (Zhang et al., 2022a). Therefore, reducing N fertilizer application, seeking economical and effective N fertilizer management strategies, improving the structure of saline and alkaline soils, and increasing crop yields are the primary objectives in agroecology and agricultural production (Chang et al., 2024).

Crop production is limited by high soil salinity, which reduces the water-extraction capacity of roots and has a devastating effect on plant metabolism. In addition, high soil salinity disrupts cellular homeostasis and results in the uncoupling of major physiological and biochemical processes (Yang et al., 2020). A high salt content in saline soils results in toxic ionic effects on soybeans and reduces soil water potential, making water and nutrients uptake difficult by soybeans (Hochberg et al., 2017). In addition, excessive accumulation of salts can impact soil nutrient transformation reactions, reducing the availability of essential nutrients in the soil. This can inhibit nutrient uptake during soybean growth and development, ultimately affecting yield (Gilliham et al., 2017). Research has shown that biochar has strong potential for ameliorating saline soils (O'Laughlin and McElligott, 2009). Biochar has a porous structure with a large specific surface area; therefore, it can increase soil permeability and regulate soil pH (Ge et al., 2018). In addition, biochar has strong water absorption and ion adsorption capacity, which is favorable for water and fertilizer retention, the regulation of soil water and salt distribution, and the generation of a suitable soil environment for crop growth (Du et al., 2023; Hu et al., 2024). Laird et al. (2010a) showed that the application of biochar to soil significantly increased the overall porosity soil and reduced soil nutrient leaching. Gao et al. (2023) concluded that the pore structure of biochar enhanced the water-holding capacity of the soil and reduced the vertical transport of water in the soil, which in turn affected the conversion, transport and vertical distribution of N. Chhabra (2005) showed that saline soils accumulate salt from the soil surface to 30 cm. Thus, tillage should be considered when biochar use is coupled with N fertilizer application to improve saline soils. Tillage is the main approach to increase the depth of biochar incorporation in soil. With proper tillage, soil fertility is indirectly enhanced through changes in the topographical and elevation characteristics of saline soil microregions, through the regulation of the structure and porosity of surface saline soils and through the promotion of salt leaching (Zhang et al., 2022b). Ding et al. (2021) showed that deep tillage was effective at reducing soil salinity and increasing water use efficiency in soda-dry fields; it significantly improved the physicochemical properties of soda-salt soils.

Through proper tillage practices, saline soil environments can be improved, the spatial and temporal distribution of soil water and salts can be regulated, and the nutrient content in soil can be increased (Wright et al., 2007).To date, few studies have been conducted on improving soda saline soils in black soil areas with N reduction and biochar application. Existing studies have mainly been focused on the amount of biochar applied, with the effect of the depth of biochar application on the soil environment largely overlooked. Therefore, in this study, the effects of biochar application depth and N fertilizer reduction on the soda saline soil environment and on soybean growth were investigated in a black soil area.

## 2 Materials and methods

### 2.1 Experimental material

The biochar added in this experiment was made from corn stover burned at 500°C under anaerobic conditions. Its basic physicochemical properties were as follows: particle size of 1.5-2.0 mm, pH value of 9.14, carbon content of 70.38%, total N content of 1.53%, sulfur mass fraction of 0.78%, hydrogen mass fraction of 1.68%, and ash content of 31.8%. The saline soil used for the experiment was collected from the 0-80 cm soil layer of the field test site. The main soil type was saline soil, the organic matter in the soil was 34.53 g/kg, the alkaline dissolved N was 177.24 mg/kg, the available phosphorus was 82.31 mg/kg, the available potassium was 160.22 mg/kg, and the soil salts were predominantly NaHCO_3_ and Na_2_CO_3_, with a pH mostly above 8.

### 2.2 Experimental methods

**Figure 1:**
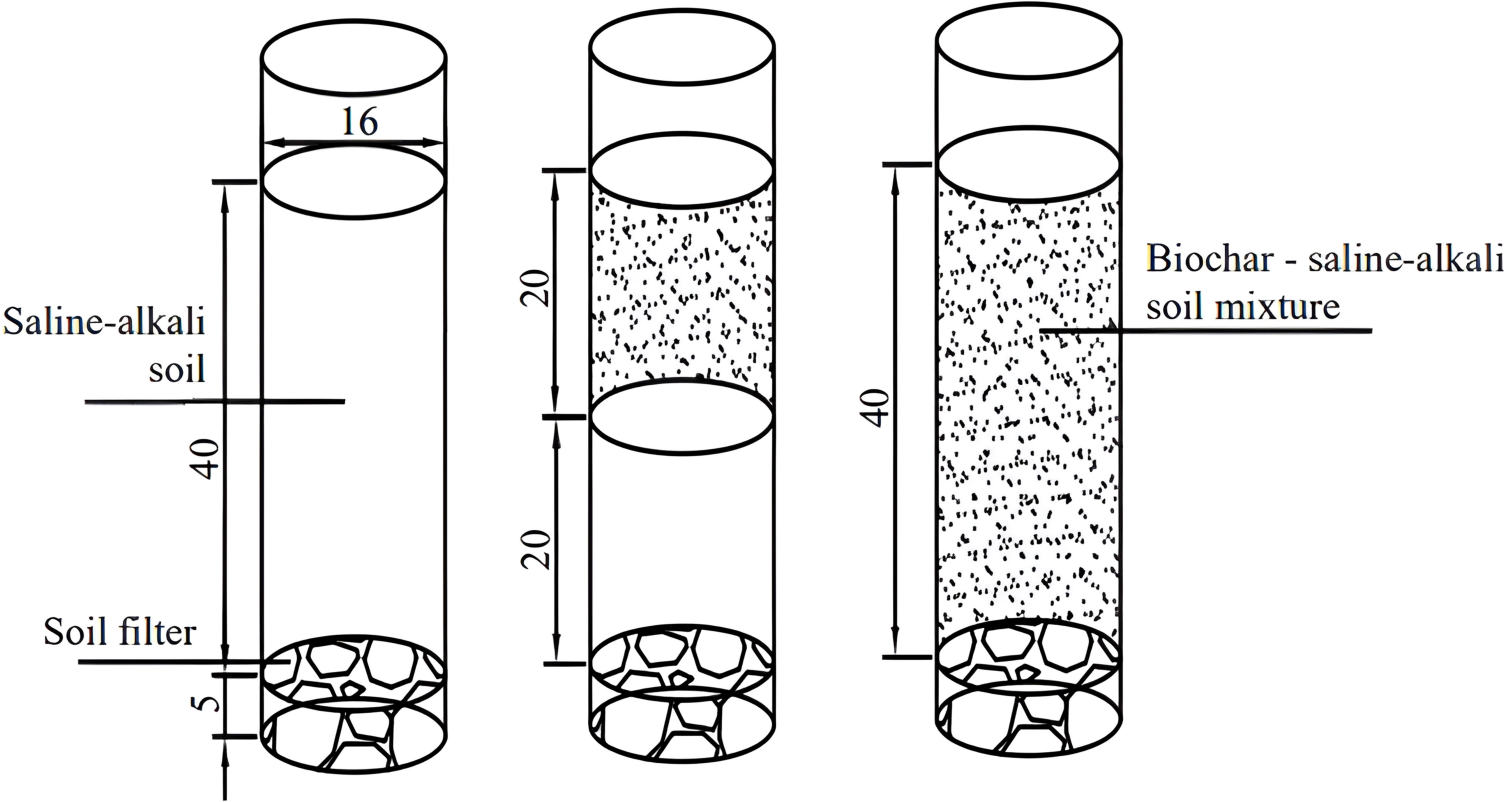
Schematic of soybean planting layout.

A pot experiment was conducted in PVC pipes with a diameter of 16 cm and a height of 55 cm, the volume of the PVC pipes was 1.1 L and the experimental crop was soybean (soybean variety *Dongsheng No. 1*). After the soil had been thoroughly air-dried and sieved, the tubes were filled with soil, and a 5 cm soil filter was placed at the bottom of each tube to prevent nutrient runoff. The weight of soil used and the amount of biochar applied were calculated based on the soil bulk weight and the volume of the PVC pipes. The pipes were filled with soil, which was compacted in layers according to the experimental design to ensure consistency with the field soil density. The experiment included seven treatments: two biochar application levels, 0 and 4.5 t/hm^2^ (biochar/soil); two biochar application depths, 0-20 cm (H1) and 0-40 cm (H2); and two N application levels, conventional N application and 15% N reduction. Conventional fertilizer application was calculated as 55 kg/hm^2^ of coated urea (N 37%), 150 kg/hm^2^ of diammonium phosphate (N 18%, P_2_O_5_ 46%), and 50 kg/hm^2^ of potassium sulfate (K_2_O 50%). The control treatment (CK) consisted of no N fertilizer and no biochar application. Fertilizer was applied immediately before sowing, and each treatment had three replicates (**Figure 1** was schematic of soybean planting layout and **Table 1** was nitrogen fertilizer and biochar application for each treatment in the pot experiment).

**Table 1:** N fertilizer and biochar application for each treatment in the pot experiment.

Treatments	Experimental design	Fertilizer consumption (g)			Dry soil application (kg)	Biochar application (kg)
		N fertilizer (g)	Phosphate fertilizer (g)	Potassium fertilizer (g)		
CK	No biochar applied no N fertilizer applied	0	1.61	0.54	11.7	0
B0N0	No biochar applied conventional N application	0.6	1.61	0.54	11.7	0
B0N1	No biochar applied 15% N reduction	0.33	1.61	0.54	11.7	0
B1H1N0	Shallow application of biochar (0-20 cm) conventional N application	0.6	1.61	0.54	11.34	0.36
B1H1N1	Shallow application of biochar (0-20 cm) 15% N reduction	0.33	1.61	0.54	11.34	0.36
B1H2N0	Deep application of biochar (0-40 cm) conventional N application	0.6	1.61	0.54	11.34	0.36
B1H2N1	Deep application of biochar (0-40 cm) 15% N reduction	0.33	1.61	0.54	11.34	0.36

According to the saturated soil water content, the pots were flooded with 3 L of water before the start of the experiment to ensure that they were well watered, and water consumption was measured daily. According to the inner diameter of the soil column, four sowing points in the shape of a square were set up in each pot, spaced 6 cm apart; each point was sown with 2-3 seeds, and one soybean plant was retained in each hole after the emergence of the seedlings. The soil columns were weighed, and the soil moisture content was determined at 9:00 a.m. each day. The amount of water to be added was determined by measuring the weight and moisture content of the soil in CK, and the average weight and moisture content of the soil was taken accordingly (Bittelli, 2011). When the soil moisture content of the pots was below or close to the design criteria, water was added to the measuring cups, and the amount of water added was recorded. The lower limit of irrigation was 60% of the field water holding capacity, and the upper limit was 90% of the field water holding capacity.

The soybean plants grew vigorously during the flowering period, so the flowering period was used as the experimental period (days 50 to 75 post-sowing). Plant samples and soil samples from the 0-20 cm soil layer and the 20-40 cm soil layer were collected during the soybean flowering period, and relevant indexes were measured. The soil was subsequently replenished in time at the location where the soil was collected after the soil was collected.

### 2.3 Ammonium N and nitrate N contents in the soil

Fresh soil samples were collected at the flowering stage and sieved through a 2 mm sieve. Soil samples (10.0 g) were accurately weighed, added to 100 ml of 1 mol/L KCl solution, shaken for 60 min, and filtered, and then the filtrate was analyzed by a Bran + Luebbe AA3 instrument (AutoAnalyzer-III, SEALAnalytical, German) (Chaganti and Crohn, 2015).

### 2.4 K^+^, Na^+^, Ca^2+^ and Mg^2+^ ions in soil

After the soil samples were allowed to dry naturally at the flowering stage, they were sieved through a 0.15 mm sieve, and 5.00 g of soil sample was weighed, added to 50 ml of 1 mol/L ammonium acetate solution (pH=7) and shaken for 30 min. The samples were analyzed by atomic absorption spectroscopy (AAS) (Chaganti and Crohn, 2015).

### 2.5 Sodium adsorption ratio and exchangeable sodium saturation percentage in soil

The SAR was calculated as


(1) $$\begin{array}{c} \text{SAR=}\frac{{\text{C}}_{{\text{Na}}^{+}}}{1/2{\left[{\text{C}}_{{\text{Ca}}^{2+}}+{\text{C}}_{{\text{Mg}}^{2+}}\right]}^{1/2}} \end{array}$$

where C denotes the corresponding ion concentration in mmol/L (Lotfollahi et al., 2023)

The ESP was calculated as


(2) $$\begin{array}{c} \text{ESP=}\frac{100\left(-0.0126+0.01475\times \text{SAR}\right)}{1+\left(-0.0126+0.01475\times \text{SAR}\right)} \end{array}$$

### 2.6 Soybean leaf total N value

Flowering leaves were removed, digested with concentrated H_2_SO_4_-H_2_O_2_ and measured by the Bran+Luebbe AA3 method) (Chaganti and Crohn, 2015).

### 2.7 Soybean yield and its components

When the soybean plants reached the full maturity stage (R8), the yield, soybean grain weight per plant, and number of grains per plant were measured.

### 2.8 Nitrogen fertilizer agronomic efficiency

The N fertilizer agronomic efficiency (NAE) was calculated as (Huang et al., 2021)


(3) $$\begin{array}{c} \begin{array}{l} \text{Nitrogen fertilizer agronomic efficiency(kg/kg)}\\ =\frac{\text{soybean yield with nitrogen application - soybean yield without nitrogen application}}{\text{nitrogen application}} \end{array} \end{array}$$

### 2.9 Partial factor productivity of N

The partial factor productivity of N was calculated as (Huang et al., 2021)


(4) $$\begin{array}{c} \text{Partial factor productivity of N(kg/kg)=}\frac{\text{yield}}{\text{nitrogen application}} \end{array}$$

### 2.10 Relative chlorophyll content

SPAD was determined using a MultispeQ multifunctional chlorophyll meter, in which fully expanded and inverted tertiary functioning leaves at the uppermost part of soybean plants were selected for measurement at the flowering stage, and the average values were taken after measurement (Tian et al., 2022).

### 2.11 Photosynthesis rate and water use efficiency of soybean leaves

Photosynthesis rates were measured at the flowering stage by a Li-6400 portable photosynthesis analyzer (LI-COR Inc, Lincoln, USA). The time of measurement was 9:00-11:00 a.m. on a sunny and cloudless day, and the inverted trifunctional leaves of the uppermost part of the soybean plants were selected for the measurement. The net photosynthetic rate (*A_N_*), transpiration rate (*T_r_*), intercellular CO_2_ concentration (*C_i_*), and stomatal conductance (*g_s_*) of the soybean leaves were measured, and the average values were taken (Liu et al., 2024). The WUE was calculated as *A_N_/g_s_* (Ma et al., 2021).

### 2.12 Total leaf water potential and water content of soybean

Total leaf water potential (Ψ_leaf_) was measured using a pressure chamber (Model 3000F01H12G2P40, Soil Moisture Equipment Corp., Santa Barbara, CA, USA). Fully expanded leaves were harvested and then immediately wrapped in aluminum foil and dropped into liquid nitrogen container. Thereafter, the samples were stored at 80°C until analysis of osmotic potential. Water content (WC) was calculated as


(5) $$\begin{array}{c} \text{WC=}\frac{\text{FW-DW}}{\text{FW}}\times 100\% \end{array}$$

where FW denotes the Fresh weight of soybean concentration in g. DW denotes the Dry weight of soybean concentration in g.

### 2.13 Nitrogen Use Efficiency

Nitrogen Use Efficiency (NUE) was calculated as NUE is commonly defined as vegetative yield per unit of N available to the crop (g DW g^−1^ N) (Rosales et al., 2020).

### 2.14 Statistical analysis

Results are presented as the mean ± standard deviation of three independent experiments. Normality of data set was verified by Klomogorov-Smirnov test. Statistical differences were determined by one-way analysis of variance followed by Tukey’s test in SPSS version 26.0 (IBM, Armonk, NY, USA), with p< 0.05 considered significant. Two-way analysis of variance was used to test the effects of biochar, N fertilizer, and their interactions; ns, P > 0.05; *, P< 0.05; **, P< 0.01. ***, P< 0.001. All figures were prepared using Origin 2024 (OriginLab, USA).

## 3 Results

### 3.1 Effects of biochar and its application depth coupled with N fertilizer reduction on K^+^, Na^+^, Ca^2+,^ and Mg^2+^ ions in the soil

**Figure 2:**
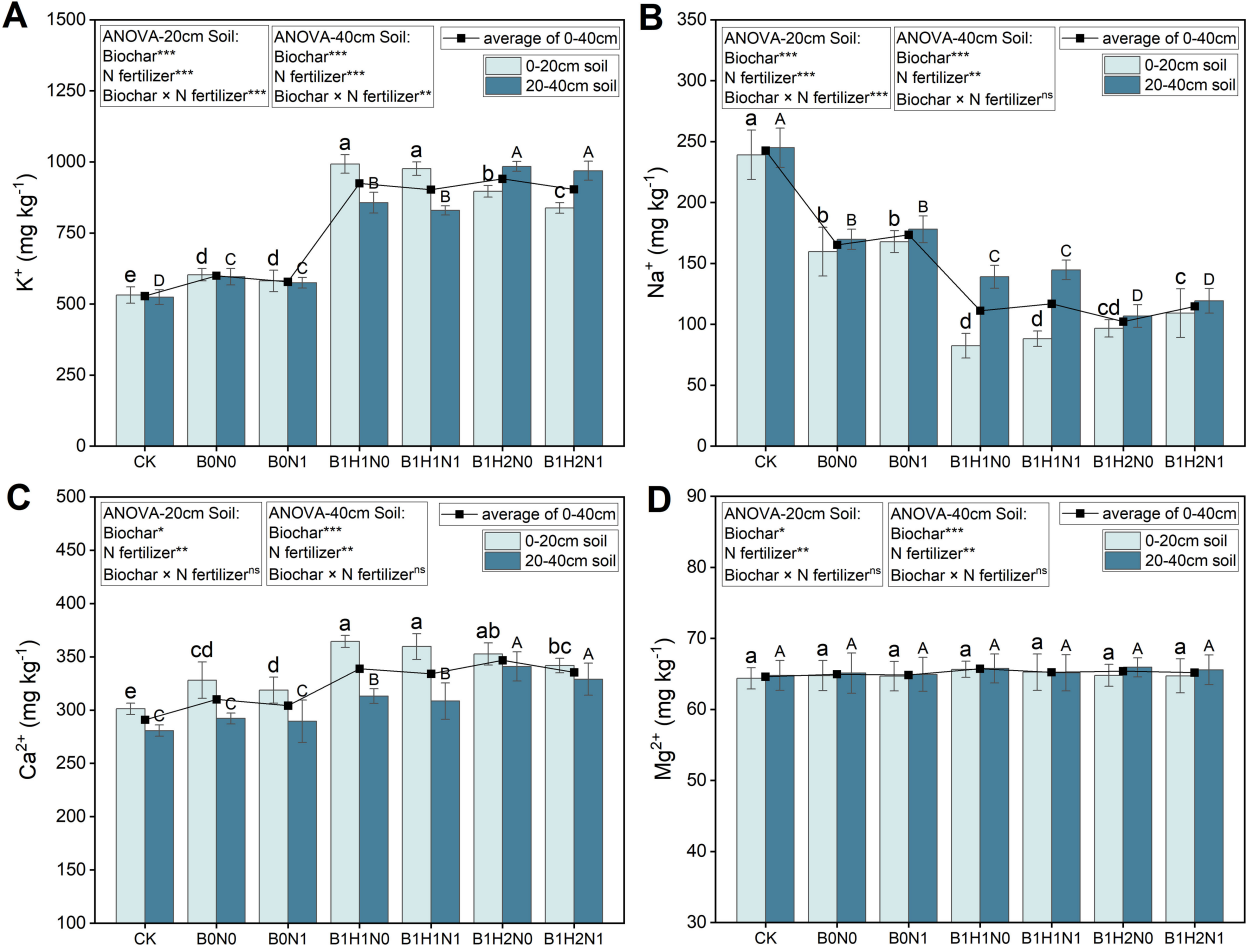
K^+^ **(A)**, Na^+^ **(B)**, Ca^2+^ **(C)**, and Mg^2+^ **(D)** contents in the 0-20 cm and 20-40 cm soil layers. Two biochar application levels, 0 (B0) and 4.5 t/hm^2^ (B1); two biochar application depths, 0-20 cm (H1) and 0-40 cm (H2); and two N application levels, conventional N (N0) application and 15% N reduction (N1). Homogeneous group statistics were calculated through ANOVA tests, where mean values with different letters are significantly different according to Tukey’s test. Summary of two-way ANOVAs for the effects of biochar, N fertilizer, and their interactions, ns, P > 0.05 *, P< 0.05; **, P< 0.01. ***, P< 0.001.

The changes in K^+^, Na^+^, Ca^2+^, and Mg^2+^ contents during the flowering stage of soybean for each treatment are shown in **Figure 2**. In the 0-40 cm soil layer, reduced N fertilization decreased the K^+^ and Ca^2+^ contents and increased the Na^+^ content; in contrast, the application of biochar significantly increased the soil K^+^ and Ca^2+^ contents and significantly reduced the Na^+^ content. In the coupled mode, the K^+^, Ca^2+^, and Na^+^ contents differed significantly from those in B0N0. However, the effects of N application and biochar application on the Mg^2+^ content were not significant. Under the same N application treatment, increasing the depth of biochar application increased the K^+^ and Ca^2+^ contents and decreased the Na^+^ content in the 0-40 cm soil layer. In addition, the effects of biochar application depth on the K^+^, Ca^2+^, and Na^+^ contents differed among the different soil layers. Under the same N application treatment, the K^+^ and Ca^2+^ contents decreased with increasing biochar application depth, and the Na^+^ content increased with increasing biochar application depth in the 0-20 cm soil layer. Compared to B0N0, B1H1N1 and B1H2N1 showed increases in K^+^ content of 61.87% and 38.97%, increases in Ca^2+^ content of 9.61% and 4.19%, and decreases in Na^+^ content of 44.80% and 31.65%, respectively. In contrast, in the 20-40 cm soil layer, the K^+^ and Ca^2+^ contents increased with increasing biochar application depth, and the Na^+^ content decreased with increasing biochar application depth. Compared to B0N0, B1H1N1 and B1H2N1 showed increases in K^+^ content of 39.09% and 62.54%, increases in Ca^2+^ content of 5.56% and 12.58%, and decreases in Na^+^ content of 14.84% and 29.76%, respectively.

### 3.2 Effects of biochar and biochar application depth combined with N fertilizer reduction on the soil sodium adsorption ratio, exchangeable sodium saturation percentage, and Na^+^/K^+^ ratio

**Figure 3:**
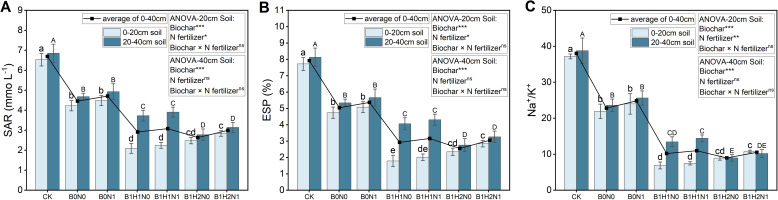
SAR **(A)**, ES P**(B)**, and Na^+^/K^+^ **(C)** in the 0-20 cm and 20-40 cm soil layers. Two biochar application levels, 0 (B0) and 4.5 t/hm^2^ (B1); two biochar application depths, 0-20 cm (H1) and 0-40 cm (H2); and two N application levels, conventional N (N0) application and 15% N reduction (N1). Homogeneous group statistics were calculated through ANOVA tests, where mean values with different letters are significantly different according to Tukey’s test. Summary of two-way ANOVAs for the effects of biochar, N fertilizer, and their interactions ns, P > 0.05, *, P< 0.05; **, P< 0.01. ***, P< 0.001. SAR, Sodium adsorption ratio, ESP, exchangeable sodium saturation percentage.

The SAR, ESP, and Na^+^/K^+^ ratio at the flowering stage of soybean plants in each treatment are shown in **Figure 3**. In the 0-40 cm soil layer, N fertilizer application increased the SAR, but biochar application significantly reduced the SAR. In the coupled mode, the SAR was significantly lower than that in B0N0. The incorporation of biochar into deep soil layers reduced the SAR in the 0-40 cm soil layer under the same N application treatment. In addition, the depth of biochar application had different effects on the SAR in the different soil layers. Under the same N application treatment, the SAR increased with increasing biochar application depth in the 0-20 cm soil layer and decreased with increasing biochar application depth in the 20-40 cm soil layer. Compared to those of B0N0, the SARs of B1H1N1 and B1H2N1 were reduced by 46.68% and 32.70% and by 16.55% and 32.85%, respectively, in the 0-20 cm soil layer and 20-40 cm soil layer. In addition, the trends in ESP and Na^+^/K^+^ at the soybean flowering stage in each treatment were similar to those in the SAR treatment.

### 3.3 Effects of biochar and its application depth coupled with N fertilizer reduction on nitrate N and ammonium N content

**Figure 4:**
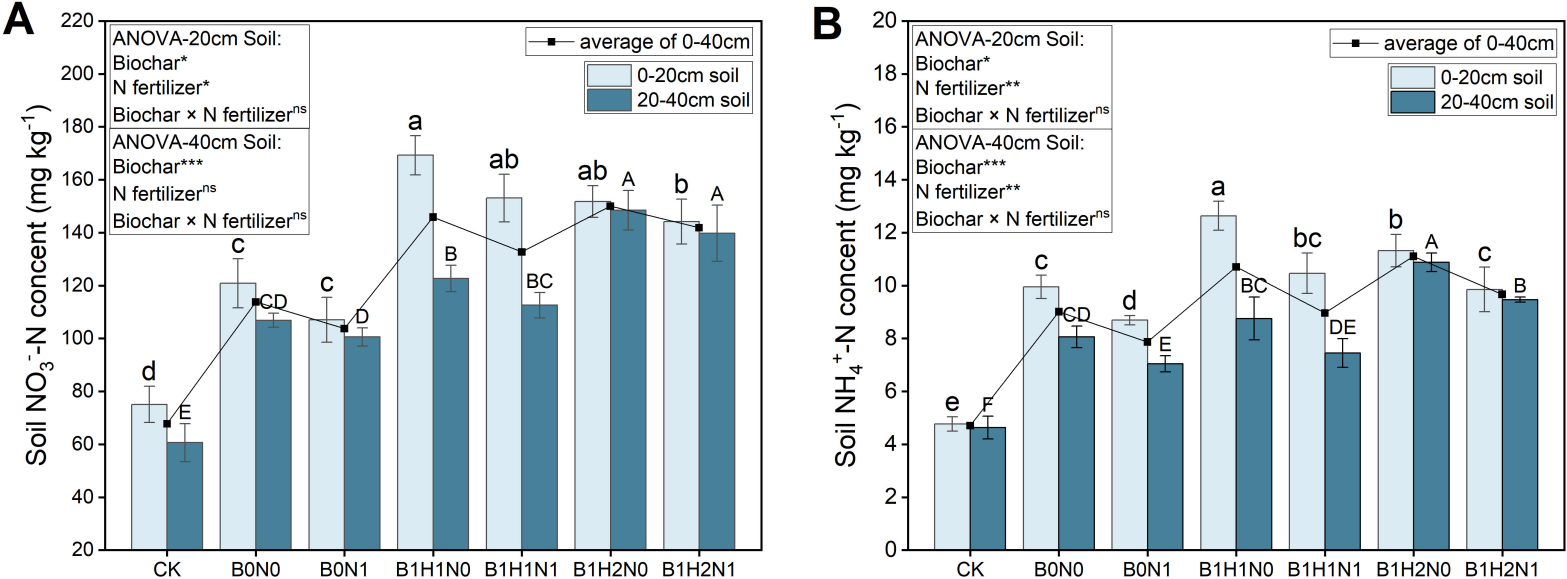
Nitrate N **(A)** and ammonium N **(B)** contents in the 0-20 cm (left) and 20-40 cm (right) soil layers. Two biochar application levels, 0 (B0) and 4.5 t/hm^2^ (B1); two biochar application depths, 0-20 cm (H1) and 0-40 cm (H2); and two N application levels, conventional N (N0) application and 15% N reduction (N1). Homogeneous group statistics were calculated through ANOVA tests, where mean values with different letters are significantly different according to Tukey’s test. Summary of two-way ANOVAs for the effects of biochar, N fertilizer, and their interactions ns, P > 0.05, *, P< 0.05; **, P< 0.01. ***, P< 0.001.

The changes in nitrate N and ammonium N in each treatment group at the flowering stage are shown in **Figure 4**. In the 0-40 cm soil layer, a decrease in the application of N fertilizer decreased the nitrate N content of the soil, while the application of biochar increased the nitrate N content. The nitrate N content in the soil significantly increased with increasing depth of biochar application. The effects of biochar application depth on nitrate N differed among the different soil layers. The nitrate N content decreased with increasing depth of biochar application in the 0-20 cm soil layer under the same N application treatment, and the nitrate N contents of B1H1N0 and B1H1N1 were greater than those of the other treatments, but the difference between the treatments was not significant. However, the nitrate N content increased with increasing depth of biochar application in the 20-40 cm soil layer. Both the B1H2N0 and B1H2N1 nitrate N contents were greater than those in the other treatments, but the difference between the treatments was not significant. Compared with those of B0N0, the nitrate N contents of B1H1N1 and B1H2N1 increased by 26.61% and 19.27% and 5.32% and 30.77% in the 0-20 cm and 20-40 cm soil layers, respectively. At the same soil depth, the trends in ammonium N and nitrate N contents in soybean plants at the flowering stage were the same among all treatments. Compared with B0N0, in the 0-20 cm soil layer, only B1H1N1 increased ammonium N by 5.13% in the N reduction with biochar treatment, and the difference was not significant. In the 20-40 cm soil layer, only B1H2N1 increased ammonium N by 17.45%, and the difference was significant.

### 3.4 Effects of biochar and its application depth coupled with N fertilizer reduction on leaf total N and nitrogen use efficiency

**Figure 5:**
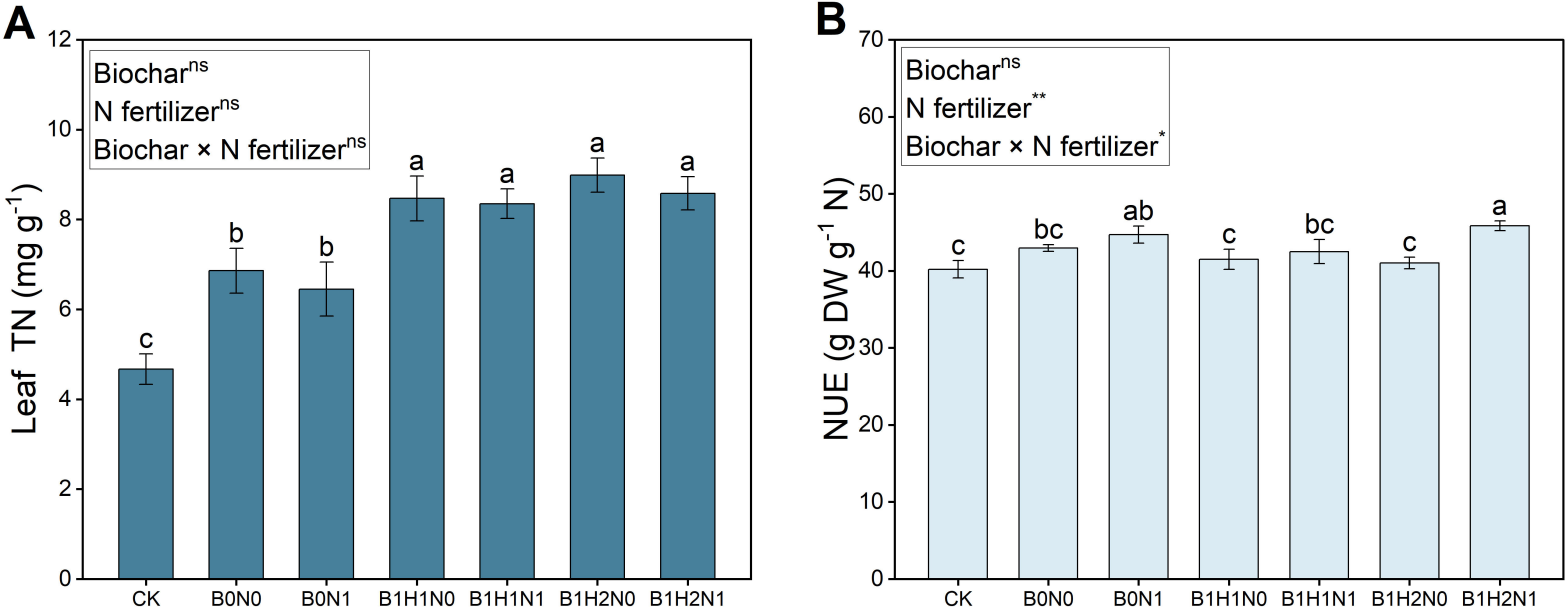
Leaf total N **(A)**, nitrogen use efficiency **(B)**. Two biochar application levels, 0 (B0) and 4.5 t/hm^2^ (B1); two biochar application depths, 0-20 cm (H1) and 0-40 cm (H2); and two N application levels, conventional N (N0) application and 15% N reduction (N1). Homogeneous group statistics were calculated through ANOVA tests, where mean values with different letters are significantly different according to Tukey’s test. Summary of two-way ANOVAs for the effects of biochar, N fertilizer, and their interactions ns, P > 0.05, *, P< 0.05; **, P< 0.01. ***, P< 0.001. Leaf TN, leaf total N. NUE, nitrogen use efficiency.

The leaf total N at the flowering stage of soybean for each treatment is shown in **Figure 5**. A decrease in N fertilizer application decreased soybean leaf total N, but biochar application increased soybean leaf total N. In the coupled mode, leaf total N was significantly greater than that in the B0N0 treatment. Under the same N application treatment, compared with shallow biochar application, deep biochar application increased soybean leaf total N by 6.14% (N0) and 2.75% (N1). Leaf total N increased by 21.72% and 25.07% in B1H1N1 and B1H2N1, respectively, compared to that in B0N0. The nitrogen use efficiency of soybean in each treatment is shown in **Figure 5B**. A decrease in N fertilizer application improved the NUE of soybean, and deep application of biochar further promoted the improvement of NUE of soybean, and compared with the CK, the NUE of the B1H2N1 was significantly increased by 14.04%.

### 3.5 Effects of biochar and its application depth coupled with N fertilizer reduction on soybean yield and its components and N fertilizer agronomic efficiency

The soybean yield and its components and NAE for each treatment are shown in **Table 2**. The N reduction treatment reduced soybean yield and NAE, while biochar application increased soybean yield and NAE. The soybean yield and NAE increased under the coupled mode. The deep application of biochar increased soybean yield and its components and NAE under the same N application treatments. Compared to B0N0, B1H1N1 and B1H2N1 increased soybean grain number by 2.56% and 7.69%, NAE by 1.28% and 33.01%, and partial factor productivity of N by 13.43% and 28.37%, respectively. In addition, among the N reduction coupled with biochar treatments, only B1H2N1 significantly increased soybean grain weight by 3.33% compared to B0N0.

**Table 2:** Soybean yield and its components and N fertilizer utilization rate.

Treatment	Number of soybean grains (pcs)	Soybean grain weight (g·pot^-1^)	Nitrogen fertilizer agronomic efficiency (kg·kg^-1^)	Partial factor productivity of N (kg·kg^-1^)
CK	27.00 ± 1.00e	5.40 ± 0.06g		
B0N0	39.33 ± 1.53c	10.21 ± 0.13d	9.39 ± 0.25d	19.95 ± 0.25e
B0N1	36.00 ± 1.00d	8.12 ± 0.05f	6.60 ± 0.18e	19.71 ± 0.11e
B1H1N0	41.67 ± 1.53bc	10.83 ± 0.15b	10.60 ± 0.34c	21.16 ± 0.29d
B1H1N1	40.00 ± 1.73bc	9.32 ± 0.07e	9.51 ± 0.29d	22.63 ± 0.17c
B1H2N0	45.33 ± 1.15a	12.17 ± 0.07a	13.22 ± 0.07a	23.78 ± 0.14b
B1H2N1	42.00 ± 1.00b	10.55 ± 0.16c	12.49 ± 0.25b	25.61 ± 0.39a

Two biochar application levels, 0 (B0) and 4.5 t/hm^2^ (B1); two biochar application depths, 0-20 cm (H1) and 0-40 cm (H2); and two N application levels, conventional N (N0) application and 15% N reduction (N1). Different lowercase letters after the data in the same column indicate significant differences between treatments (p<0.05). ‘Homogeneous group’ statistics were calculated through ANOVA tests, where mean values with different letters are significantly different according to Tukey’s test.

### 3.6 Effects of biochar and its application depth coupled with N fertilizer reduction on the photosynthetic rate

**Figure 6:**
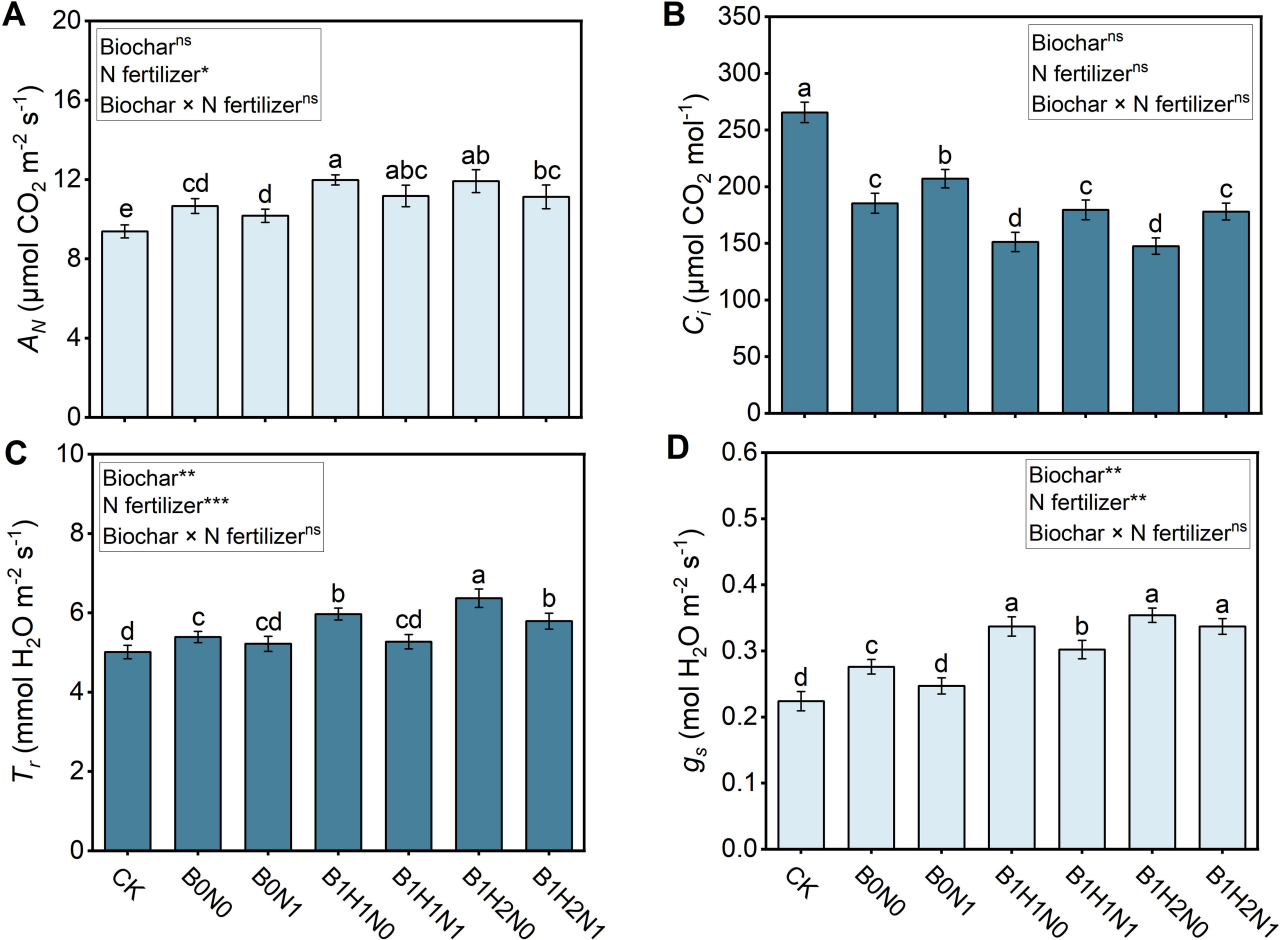
Photosynthetic rate of soybeans. *A_N_*: The net photosynthetic rate **(A)**, *T_r_:*transpiration rate **(B)**, *C_i_*: intercellular CO_2_ concentration **(C)**, *g_s_*: stomatal conductance **(D)**. Two biochar application levels, 0 (B0) and 4.5 t/hm^2^ (B1); two biochar application depths, 0-20 cm (H1) and 0-40 cm (H2); and two N application levels, conventional N (N0) application and 15% N reduction (N1). Homogeneous group statistics were calculated through ANOVA tests, where mean values with different letters are significantly different according to Tukey’s test. Summary of two-way ANOVAs for the effects of biochar, N fertilizer, and their interactions ns, P > 0.05, *, P< 0.05; **, P< 0.01. ***, P< 0.001.

The net photosynthetic rate (*A_N_*), transpiration rate (*T_r_*), stomatal conductance (*g_s_*), and intercellular CO_2_ concentration (*C_i_*) of each treatment are shown in **Figure 6**. A decrease in N fertilizer application decreased the *A_N_*, *T_r_*, and *g_s_* and increased the *C_i_* in soybean plants, while biochar application increased the *A_N_*, *T_r_*, and *g_s_* and decreased the *C_i_*. In the coupled mode, *A_N_* and *g_s_* were greater and *C_i_* was lower in the N reduction with biochar treatment than in the B0N0 treatment. Deep application of biochar further increased the photosynthetic rate. Among the N reduction plus biochar treatments, the *A_N_* of B1H1N1 and B1H2N1 increased by 4.40% and 4.78%, respectively, compared with that of B0N0, and the difference was not significant. Only the *T_r_* of B1H2N1 was significantly greater than that of B0N0, with an increase of 7.42%. Similarly, the *g_s_* of B1H1N1 and B1H2N1 increased by 9.42% and 22.10%, respectively, compared with that of B0N0, and the difference was significant. The *C_i_* of B1H1N1 and B1H2N1 decreased by 3.24% and 3.94%, respectively, compared with that of B0N0, and the differences were not significant.

### 3.7 Effects of biochar and its application depth coupled with N fertilizer reduction on relative chlorophyll content, leaf water potential, water content and water use efficiency

**Figure 7:**
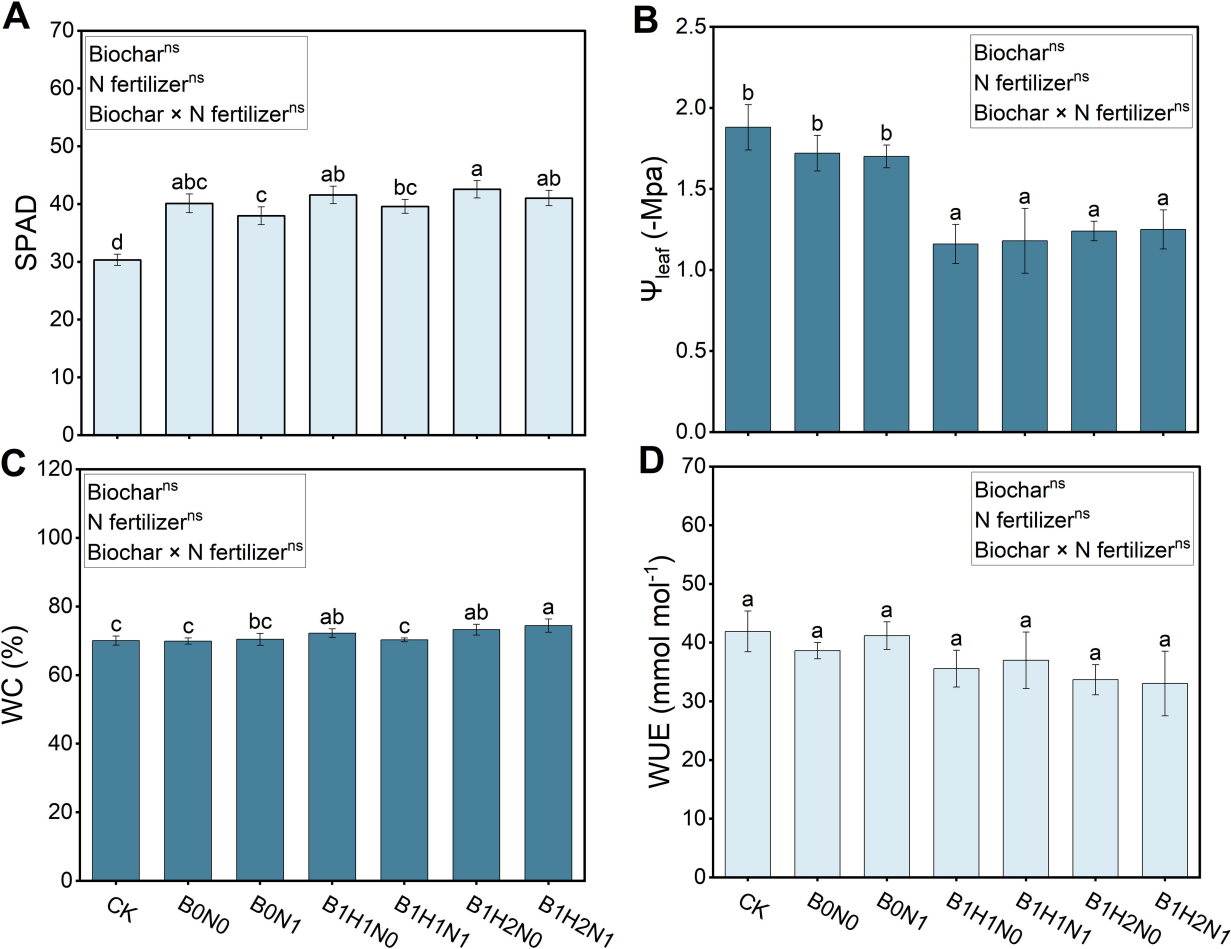
Relative chlorophyll content (SPAD) **(A)**, leaf water potential **(B)**, water content **(C)** and water use efficiency **(D)**. Two biochar application levels, 0 (B0) and 4.5 t/hm^2^ (B1); two biochar application depths, 0-20 cm (H1) and 0-40 cm (H2); and two N application levels, conventional N (N0) application and 15% N reduction (N1). Homogeneous group statistics were calculated through ANOVA tests, where mean values with different letters are significantly different according to Tukey’s test. Summary of two-way ANOVAs for the effects of biochar, N fertilizer, and their interactions ns, P > 0.05, *, P< 0.05; **, P< 0.01. ***, P< 0.001. SPAD, relative chlorophyll content; Ψ_leaf_, leaf water potential; WC, water content; WUE, water use efficiency.

The SPAD, leaf water potential, water content (WC) and water use efficiency (WUE) of each treatment are shown in **Figure 7**. A decrease in N fertilizer application decreased the SPAD and increased the leaf water potential, while biochar application increased the SPAD and WC, but decreased the leaf water potential and WUE. In the coupled mode, the SPAD and WC increased, the leaf water potential and WUE decreased. The deep application of biochar increased the SPAD and WC and decreased leaf water potential under the same N application treatment. Compared with B0N1, SPAD increased by 8.12% and WC increased by 5.67% in B1H2N1, and the leaf water potential decreased by 30.59% and 26.47% in B1H1N1 and B1H2N1, respectively, in response to biochar treatment. In addition, compared with CK, B1H2N1 increased the SPAD by 35.28%, the WC by 6.2%, and decreased the water potential by 33.51%. Notably, biochar application reduced soybean water use efficiency, but the difference was not significant, indicating that different depths of biochar application did not negatively affect soybean WUE.

## 4 Discussion

### 4.1 Effects of biochar and its application depth coupled with N fertilizer reduction on soil salt ion concentrations

Soil water and salt distribution is the main soil environmental factor limiting crop growth in saline soils, and excessive N application may cause secondary salinization. Balancing soil fertility improvement with the risk of triggering secondary salinization is highly important for improving saline soils (Ferreyra et al., 1997; Sahab et al., 2021). The improvement of saline soils by biochar mainly occurs due to the porous structure, large specific surface area, and strong adsorption capacity of biochar (Lan et al., 2024). In the experiment, it was found that the application of biochar significantly increased the content of K^+^ and Ca^2+^ and significantly reduce the content of Na^+^ in the soil under decreases in N fertilizer application (**Figure 2**), which was consistent with the conclusions of Xu et al. (2013). Due to the rich pore structure of biochar, it not only adsorbs Na^+^ from the soil but also releases K^+^ and Ca^2+^ into the soil (Xu et al., 2013). On the other hand, the organic matter accumulated in biochar could serve as a nutrient source for microorganisms in the soil, promoting the growth and activity of microbial communities and increasing the activity and biomass of microorganisms in the soil. These microorganisms can decompose organic matter, thereby increasing the soil ion exchange capacity (Frac et al., 2023). Biochar also increased the soil water content and reduced the concentration of harmful salt ions in the soil (Gou and Qu, 2018). In addition, Ca^2+^ released from biochar combines with organic acids in the soil to transform harmful sodium salts into harmless calcium salts, thus significantly reducing the Na^+^ content in the soil (Farhangi-Abriz and Torabian, 2018; Yang et al., 2019). However, the effect of biochar application on Mg^2+^ in the soil was not significant. Hailegnaw et al. (2019) concluded that due to the high Mg^2+^ content in saline soils, the release of additional Mg^2+^ ions from biochar had little effect on the overall Mg^2+^ content of the soil.

The deep application of biochar enhanced the ameliorating effect of biochar on salt ions. Under the same level of N application, the deep application of biochar increased the K^+^ and Ca^2+^ contents and decreased the Na^+^ content in the 0-40 cm soil layer (**Figure 2**). Gou and Qu (2018) concluded that a Deep application of biochar shifted the biochar-soil interface downward and improved water infiltration, thus promoting ion exchange in the 0-40 cm soil layer as a whole and making the ion distribution in the 0-40 cm soil layer more uniform, and thus improving the soil environment better. In addition, Nguyen et al. (2017) showed that under moderate biochar application, the K^+^ and Ca^2+^ contents were positively correlated with biochar application, and the Na^+^ content was negatively correlated with biochar application. As a result, the K^+^ and Ca^2+^ contents were greater in the shallow biochar treatment than in the deep biochar treatment, and the Na^+^ content was lower than that in the deep biochar treatment in the 0-20 cm soil layer. In the 20-40 cm soil layer, the trend was reversed, which was due to the fact that the deeper biochar treatment improved the 20-40 cm soil layer, and the biochar concentration was higher than that of the shallow biochar treatment, which had a better soil improvement ability than the shallow biochar treatment, resulting in the deeper biochar treatment having a higher content of K^+^ and Ca^2+^ than that of the shallow biochar treatment, and a lower content of Na^+^ than that of the shallow biochar treatment. Besides, the K^+^ and Ca^2+^ contents in the 20-40 cm soil layer were greater in the H1 treatment with biochar than in the N-only treatments at the same N application level. (Laird et al., 2010a) concluded that the difference in the porosity of the charcoal–soil interface led to waterlogging, which allowed the nutrients that leached down to accumulate in lower soil layers, making the ion content in lower soil layers higher than that in the upper soil layer.

### 4.2 Effects of biochar and its application depth coupled with N fertilizer reduction on N content in soil

Nitrogen is an indispensable mineral nutrient for crop growth and development, and fertilizer application can enhance soil fertility in saline soil. The application of biochar combined with fertilizer can significantly enhance soil fertility (Dong et al., 2022). Liu et al. (2022) showed that the application of biochar significantly increased the N content of soil under reduced N fertilizer application, and the experimental results of the current study were consistent with this conclusion. Biochar is made from the pyrolysis of biomass such as straw, which contains N, so biochar can be applied to the soil as a “N source” to enhance soil fertility (Chen et al., 2019). On the other hand, due to the porous structure and strong adsorption capacity of biochar, it can not only release fertilizers slowly but also reduce the volatilization of ammonia and the leaching of N during N cycling. The abundant functional groups present in biochar can form organic inorganic complexes with mineral and organic carbon in the soil to ensure a continuous supply of N (Beesley et al., 2011). Moreover, the application of biochar significantly reduced the Na^+^ ion content in the soil and enhanced the microbial activities in the soil, thus promoting the growth of nitrifying bacteria and nitrification (Peng et al., 2022). The increase in the N content of the soil in turn decreased the Na^+^ content. Research by Kawakami et al. (2010) showed that an increase in the N content in the soil increased the NH_4_^+^ content, which competed with Na^+^ and reduced the Na^+^ content. On the other hand, the increased N content in the soil promoted the competitive replacement of Na^+^ with Ca^2+^ in saline soils to form precipitates, alleviating the accumulation of salts in the soil. Moreover, Farhangi-Abriz and Torabian (2018) discovered that an increase in the N content in soil promoted the formation of water-stable aggregates, which adsorbed free Na^+^ from saline soils, resulting in a decrease in the Na^+^ content.

Compared with shallow biochar application, deep application of biochar further increased the N content of the 0-40 cm soil layer (**Figure 4**). The deep application of biochar altered the soil bulk density of the 0-40 cm soil layer, which in turn affected the soil microbiological properties (Zhang et al., 2021). A study by Ding et al. (2017) showed that deep application of biochar increased soil aeration and water retention and significantly enhanced the vigor of nitrifying bacteria in the soil, which increased the inorganic N content of deeper soil, thus leading to an overall increase in inorganic N content. On the other hand, deep application of biochar reduced the Na^+^ content and provided a low-salt environment for nitrifying bacteria in the deep soil, resulting in enhanced microbial activity and increased N content (Peng et al., 2022). The ability of biochar to release fertilizers slowly also allowed for the continuous supply of N from deeper soil layers and prevented N leaching from the soil by reducing the transfer and transport of nutrients through the vertical movement of soil moisture (Sim et al., 2021; Hamada et al., 2023). Clough et al. (2010) showed that biochar application influenced the nitrification process of microorganisms in the soil, and an appropriate increase in the amount of biochar applied could promote nitrification. In the present study, the inorganic N content in the 0-20 cm soil layer in the shallow biochar treatment was greater than that in the deep biochar treatment under the same N application treatment. Prommer et al. (2014) concluded that due to a higher biochar content in the 0-20 cm soil layer in the shallow biochar application treatment than in the deep biochar application treatment, nitrification was promoted in the 0-20 cm soil layer, resulting in an increase in the soil inorganic N content.

### 4.3 Effects of biochar and its application depth coupled with N fertilizer reduction on the physiological indexes of soybean growth

Nitrogen is an essential nutrient for crop growth and is involved in the synthesis of enzymes, proteins and amino acids required for photosynthesis (Chen et al., 2021). The influence of N application on the photosynthetic rate was mainly realized by regulating the leaf N content, and the SPAD increased with increasing leaf N content (Li et al., 2013). Moreover, a relatively high SPAD can delay leaf senescence and significantly extend the effective duration of photosynthesis, which in turn increases the photosynthetic rate of soybean plants and promotes the accumulation of photosynthetic products in the crop (Xu et al., 2019). The production characteristics of soybean dry matter are the outcome of the accumulation and distribution of photosynthetic products across various plant organs. An enhancement in photosynthetic capacity contributes to the accumulation of photosynthates, which in turn leads to an increase in the total dry matter accumulation in the soybean population (Luo et al., 2022). In the present study, the application of biochar improved photosynthesis and promoted soybean growth under reduced N fertilization (**Figure 6**). The application of biochar improved the level of carbon and N metabolism in soybean leaves and increased leaf soluble protein and nitrate reductase activity, which increased crop N uptake, increased the ability of the leaves to maintain a relatively high SPAD value, and increased the photosynthetic capacity of soybean plants, which led to increased soybean yields and biomass (Huang et al., 2014; Jabborova et al., 2022).

In addition, the application of biochar affected the soil environment in which the soybeans were grown. The rich porous structure of biochar not only adsorbs harmful Na^+^ ions in the soil but also increases the content of K^+^ and Ca^2+^ ions (**Figure 2**), thereby reducing the SAR, ESP, and Na^+^/K^+^ (**Figure 3**), and provided a low-salt environment for soybean root growth, resulting in decreased resistance to root growth, which was favorable for soybean root growth and biomass formation (Sandhu et al., 2017). On the other hand, due to the unique pore structure of biochar, it not only releases fertilizer slowly but also promotes the activities of nitrifying bacteria in the soil to release additional inorganic N for crop use (An et al., 2020). Moreover, biochar enables the water in the soil to be better conserved, ensuring water supply for soybean roots during growth and development (Huang et al., 2024). Furthermore, the special structure of biochar, characterized by its porous and low bulk density, effectively improves the physical environment of the soil, providing favorable conditions for root penetration and expansion. This significantly enhances the growth condition of the root system, increases the total root length during the growing period, and boosts root activity, thereby enhancing the ability of the crop to absorb water and nutrients (Ruan and Wang, 2024). Consequently, soybean plants treated with biochar exhibit higher water content and biomass compared to those treated with nitrogen alone. NUE is commonly defined as vegetative yield per unit of N available to the crop (g DW g^−1^ N; Rosales et al., 2020), therefore, due to the greater biomass of soybeans treated with biochar, this leads to an increase in soybean NUE.

This experiment found that although the application of biochar increases the net photosynthetic rate and stomatal conductance of soybeans, it decreases the WUE, which is defined as the *A_N_* to *g_s_* (Ma et al., 2021). Due to biochar’s ability to enhance soil water retention, the higher soil moisture content may facilitate easier water uptake by the plant roots, thereby maintaining a sufficient water status within the plant and reducing water stress. This allows the plant to avoid excessive stomatal closure to prevent water loss, resulting in increased *g_s_* (Lai et al., 2024). Additionally, adequate water supply helps maintain turgor pressure in leaf cells, keeping stomata open, which facilitates CO_2_ entry and promotes photosynthesis (McAdam et al., 2011). (Yu et al., 2022) found a synergistic effect between the increase in stomatal conductance and the enhancement of net photosynthetic rate. When stomatal conductance increases, more CO_2_ enters the leaf, promoting photosynthesis and consequently increasing the *A_N_*. However, in this study, we found that the application of biochar decreased the *C_i_*, which would necessitate a further increase in *g_s_* to enhance CO_2_ uptake and maintain a relatively stable intracellular CO_2_ concentration. This might result in the promotion of *A_N_* by biochar being less than the rate of increase in *g_s_*, thereby decreasing the ratio of *A_N_* to *g_s_* and reducing WUE. However, in this experiment, the reduction in WUE due to biochar application was not statistically significant, indicating that different depths of biochar application did not negatively affect soybean WUE.

The deep application of biochar further promoted soybean growth. In the present study, under the same N application treatment, the physiological growth indexes of soybean plants under the deep biochar application treatment were greater than those under the shallow biochar application treatment. Soybean is a taproot crop, and its roots can extend into deeper soil layers (Beroueg et al., 2021). The deep application of biochar improved the soil in the 0-40 cm layer, creating a favorable soil environment for the soybean root zone and promoting soybean growth. In addition, the deep biochar treatment decreased the charcoal–soil interface of the soil column, which facilitated adequate leaching of salts and altered the composition of soil salts in the 0-40 cm layer, thus reducing stress on deep crop roots due to soil salinity and alkalinity (Laird et al., 2010b).The deep application of biochar also enhanced the water retention capacity of the deep soil and increased soil permeability, allowing the roots to have more opportunities to exchange gases with the external environment (Zhang et al., 2024). Moreover, deep application of biochar revitalized deep soil resources and significantly enhanced the fertility of deep soil, increasing the amount of N available to the root system in the deeper soil layers (Hamada et al., 2023). In addition, deep application of biochar was negatively correlated with soil leaf water potential in the experiment (**Figure 7**). Xiao et al. (2023) concluded that soil moisture affected leaf water potential more significantly than did soil salinity, that some of the roots of soybean plants at the flowering stage were shallowly rooted, and that the shallow application of biochar resulted in a greater biochar content in the shallow layer than in the deeper biochar application treatment, which made the leaf water potential of the shallow treatment greater than that of the deeper biochar application treatment.

## 5 Conclusions

The deep application of biochar enhanced the effectiveness of biochar in reducing barriers to crop growth from soil salinity. As the depth of biochar application increased, the K^+^ and Ca^2+^ contents and N use efficiency increased, while the Na^+^ content, SAR, ESP, and Na^+^/K^+^ decreased. Compared with B0N0, among the N reduction plus biochar treatments, B1H2N1 was best for improving the soda saline soil environment in the black soil area, showing significant differences in the soil indexes and N use efficiency compared with B0N0.

The deep application of biochar enhanced the ability of biochar to promote soybean growth and further increased leaf total N, NUE, *A_N_*, *T_r_*, *g_s_*, SPAD, leaf water potential, WC and soybean yield and its components. Among the treatments involving reduced nitrogen and biochar application, A 15% reduction in N combined with the application of biochar at a depth of 40 cm showed the greatest promotion of soybean growth, without exerting a negative impact on the WUE of the soybeans.

## Data Availability

The original contributions presented in the study are included in the article/supplementary material. Further inquiries can be directed to the corresponding authors.
